# Single Cell Clones Purified from Human Parotid Glands Display Features of Multipotent Epitheliomesenchymal Stem Cells

**DOI:** 10.1038/srep36303

**Published:** 2016-11-08

**Authors:** TacGhee Yi, Songyi Lee, Nahyun Choi, Hyun-Soo Shin, Junghee Kim, Jae-Yol Lim

**Affiliations:** 1 SunCreate Co. Ltd., Yangju, Gyeonggi-do, Republic of Korea; 2 Translational Research Center, Inha University College of Medicine, Incheon, Republic of Korea; 3 Department of Otorhinolaryngology-Head and Neck Surgery, Inha University College of Medicine, Incheon, Republic of Korea

## Abstract

A better understanding of the biology of tissue-resident stem cell populations is essential to development of therapeutic strategies for regeneration of damaged tissue. Here, we describe the isolation of glandular stem cells (GSCs) from a small biopsy specimen from human parotid glands. Single colony-forming unit-derived clonal cells were isolated through a modified subfractionation culture method, and their stem cell properties were examined. The isolated clonal cells exhibited both epithelial and mesenchymal stem cell (MSC)-like features, including differentiation potential and marker expression. The cells transiently displayed salivary progenitor phenotypes during salivary epithelial differentiation, suggesting that they may be putative multipotent GSCs rather than progenitor cells. Both epithelial and mesenchymal-expressing putative GSCs, LGR5^+^CD90^+^ cells, were found *in vivo*, mostly in inter-secretory units of human salivary glands. Following *in vivo* transplantation into irradiated salivary glands of mice, these cells were found to be engrafted around the secretory complexes, where they contributed to restoration of radiation-induced salivary hypofunction. These results showed that multipotent epitheliomesenchymal GSCs are present in glandular mesenchyme, and that isolation of homogenous GSC clones from human salivary glands may promote the precise understanding of biological function of *bona fide* GSCs, enabling their therapeutic application for salivary gland regeneration.

Salivary hypofunction, which commonly occurs as a result of radiation damage caused to salivary glands (SGs) by treatment of head and neck cancer, causes xerostomia, swallowing difficulty, loss of taste, oral candidiasis, and dental caries^1^. This condition leads to life-long health threats as well as significant deterioration of quality of life in patients. However, there are currently no satisfactory therapies to restore radiation-induced salivary hypofunction, which warrants new emerging treatments such as cell replacement strategies, including stem cell therapy.

We recently found that intraglandular transplantation of single cell-derived mouse clonal mesenchymal stem cells (MSCs) from bone marrow (BM) could contribute to the improvement of SG hypofunction following irradiation^2^. Another recent study revealed that systemically infused human adipose tissue-derived MSCs restored SG hypofunction^3^. However, only a few infused MSCs were successfully engrafted and differentiated into SG epithelial cells in damaged SGs, suggesting that MSCs contribute to SG regeneration in a paracrine manner, rather than transdifferentiating into SG cells. Generally, regeneration of radiation-damaged SGs necessitates considerable repopulation of glandular epithelial, endothelial, myoepithelial and neural cells, as well as SG-specific tissue stem/progenitor cells. It has been suggested that multipotent tissue-resident stem cells are responsible for the functional restoration of damaged tissue by releasing various growth factors and cytokines to stimulate tissue repair and/or by differentiating into tissue-specific cells^4^. Thus, multipotent SG-specific glandular stem cells (GSCs) have the potential for therapy to treat radiation-induced SG hypofunction.

SG-resident stem/progenitor cells, which are commonly found in small numbers, have been isolated from rodent and human SGs by sorting specific marker-expressing cells or side population cells. The therapeutic potential of SG-resident stem/progenitor cells has been evaluated by their multilineage differentiation into hepatic, pancreatic, and salivary epithelial cells^5^,^6^,^7^,^8^,^9^, as well as mesenchymal cells^10^,^11^. However, it is difficult to understand the biological properties of stem cells in depth because stem/progenitor cell populations isolated by this method are mixed and heterogeneous. Thus, single cell or clonal approaches may have the advantage of providing relative cellular homogeneity in stem cell research.

We recently isolated GSCs from mouse submandibular glands by a modified subfractionation culture method and described their stem cell properties^12^. Through this method, we easily isolated and established clonal cells from stem/progenitor cell populations. Successful isolation of mouse GSCs prompted investigation of whether multipotent GSCs could be isolated from human SGs. In the present study, we established several single colony-forming unit (CFU)-derived GSC clones isolated from human parotid glands and examined their stem cell properties and molecular characteristics. We revealed that human GSCs exhibit both epithelial and mesenchymal phenotypes, as well as multipotent differentiation potential. These epitheliomesenchymal GSCs, which expressed Lgr5 and CD90, could regenerate radiation-damaged SGs. The findings presented herein improve our biological understanding of human GSCs and the possibility of their clinical application to treat radiation-induced salivary hypofunction.

## Results

### Isolation and culture-expansion of putative clonal GSCs from human parotid glands

We attempted to isolate human SG-resident GSCs by a modified subfractionation culturing method that has been shown to be effective for isolation of highly homogenous mouse clonal GSCs^12^. We obtained a number of plastic-adherent single colonies from human parotid glands and then isolated them. Several clones were culture-expanded to establish clonal cell populations, from which we randomly selected three different clones (Clone 1, 2, and 3) and examined whether they exhibit stem cell properties as putative GSCs.

### Cell morphology and proliferation activity

**Figure 1:**
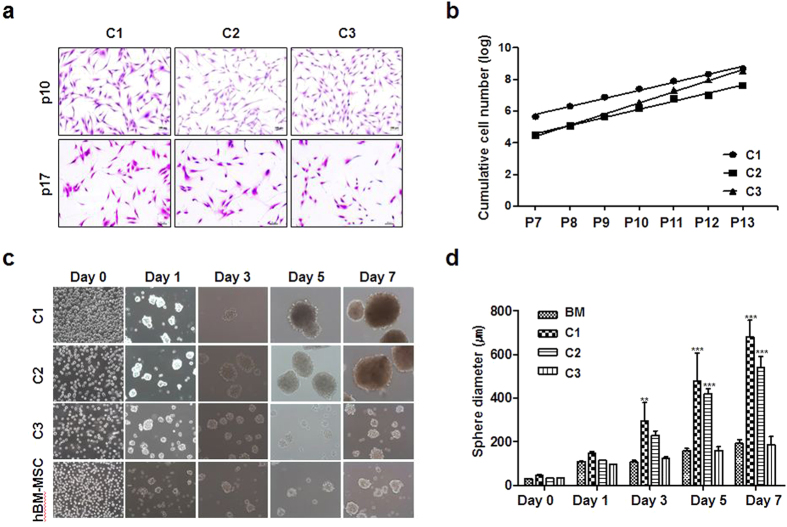
Cell morphology and proliferation of human salivary gland (SG)-derived clonal cells. (**a**) Three representative clonal cell populations (C1, C2, and C3) were isolated from SGs. The cells were stained with crystal violet for clear visualization. Each clonal population showed a fibroblast-like appearance and the morphological consistency was maintained during subculture. Magnification in each panel is 100×. (**b**) The clonal cells constantly proliferated during long-term culture and exhibited clonal variations in the proliferation activity. (**c**) Sphere-forming activity was compared. The isolated clones showed different sphere-forming activity under floating cell culture conditions. The clonal population of C1 and C2 formed larger spheres within 7 days of culture than C3. Human MSCs derived from bone marrow (BM-MSC) were compared as controls. Magnification in each panel is 200×. (**d**) Spherical diameters were measured at the indicated time points, showing the better sphere-forming activity of C1 and C2. Results are presented as the means ± SEM. One-way ANOVA, Tukey’s post hoc multiple comparison test (**p < 0.01, ***p < 0.001).

All three human clonal SG cells cultured on plastic culture plates displayed a fibroblast-like appearance under a light microscope. During subculture, the morphological consistency was maintained up to passage number 17 in a monolayer culture (Fig. 1a). The cell proliferation activity was evaluated by counting viable cell numbers in long-term culture. The clonal SG cells constantly proliferated during long-term cultivation, indicating that the clonally expanded cells are highly proliferating rather than dormant or quiescent (Fig. 1b). Clone 3 was the fastest-growing clone (Fig. 1b), and the doubling times of clones 1, 2, and 3 were 46.1 h, 51.6 h, and 37.2 h, respectively. When clonal SG cells and BM-MSCs were cultured on floating plates, they were found to form salisphere-like floating spherical aggregates (Fig. 1c). After 5 days, a significant increase in spherical diameter was observed in all of the cells. However, there were morphological differences among spheres, and the spheres generated from Clone 1 and Clone 2 were larger and denser than those from BM-MSCs and C3 (Fig. 1d). These results showed that clones derived from the same SG tissue showed differences in properties such as proliferation and sphere-forming activity.

### Differentiation potential

**Figure 2:**
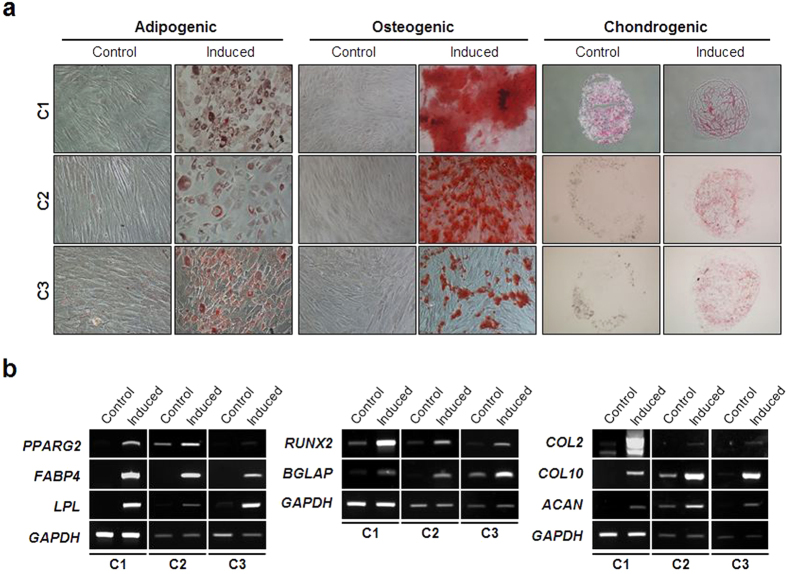
Mesenchymal differentiation potential of the SG clones. (**a**) Each clonal population was appropriately induced to differentiate into three mesenchymal cell types. At the end of each differentiation, the cells were stained with oil red O, alizarin red S, and safranin O to evaluate adipogenic, osteogenic, and chondrogenic differentiation, respectively. Magnification is 100 × and 200 × in osteogenic/chondrogenic differentiation and adipogenic differentiation panels, respectively. (**b**) Along with specific cytochemical staining, expression of molecular markers for each differentiation was also analyzed by RT-PCR.

We next explored whether human clonal SG cells possess a multipotency to differentiate into various cell types. To accomplish this, mesenchymal differentiation potential was evaluated first. When each clone was induced to adipogenic, osteogenic, and chondrogenic differentiation, it exhibited fat, bone, and cartilage phenotypes, respectively (Fig. 2a). Expression of lineage-specific molecular markers (*PPARγ2, FABP4*, and *LPL* as adipogenic markers; *RUNX2* and *BGLAP* (for osteocalcin) as osteogenic markers; and *COL2* (for type II collagen), *COL10* (for type X collagen), and *ACAN* (for aggrecan) as chondrogenic markers) was also confirmed (Fig. 2b).

**Figure 3:**
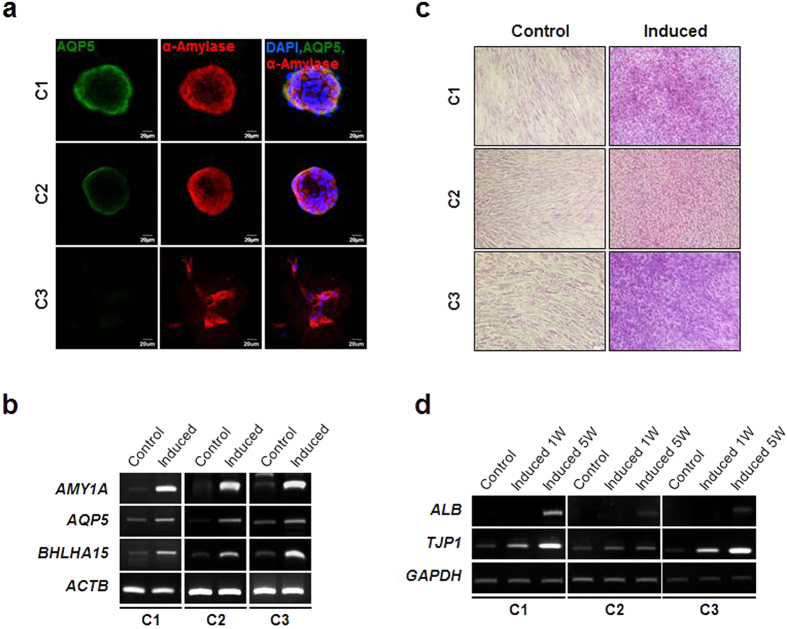
Epithelial differentiation potential. (**a**) Each SG clone was allowed to form salisphere-like cell structure followed by salivary epithelial cell differentiation as described in the methods. Salivary epithelial differentiation was evaluated by immunofluorescence staining for AQP5 and α-amylase. Scale bars represent 20 μm. (**b**) Expression of salivary epithelial differentiation-associated molecular markers including *AMY1A*, *AQP5*, and *BHLHA15* was examined by semi-quantitative RT-PCR. (**c**) To assess the multiple epithelial differentiation potential of the SG clones, each clonal population was subject to *in vitro* hepatogenic differentiation. The differentiation was evaluated by PAS staining. (**d**) Expression of hepatocyte differentiation markers including *ALB* and *TJP1* was analyzed by RT-PCR.

Next, to evaluate the epithelial differentiation potential of the SG clones, we assessed whether they were capable of differentiating into salivary epithelial cells and hepatocytes *in vitro*. For salivary epithelial differentiation, each clone was seeded onto Matrigel-coated wells and cultured in serum-free Hepato-STIM medium. The cells showed morphological changes within 3 days of induction (Fig. 3a). Specifically, they formed spherical cell aggregates that significantly expressed the salivary acinar markers, AQP-5 and α-amylase. The acinar marker genes including *AMY, AQP5*, and *BHLHA15 (also known as MIST1)* were also found to be highly induced from the differentiating cells (Fig. 3b), suggesting that they possess the potential to differentiate into salivary epithelial cells. However, there were clonal variations in sphere-forming activity and acinar marker expression. The C3 spheres exhibited relatively unstable and imperfect morphology (Fig. 3a). Lower expression of AQP5 protein expression was also evident in C3 relative to C1 and C2 (Fig. 3a).

Upon *in vitro* hepatogenic induction, three clones successfully expressed hepatocyte phenotypes. The differentiated cells were positive for PAS staining, which specifically stains glycogen in liver hepatocytes (Fig. 3c). Hepatocyte marker genes including *ALB* (encoding albumin) and *TJP1* (encoding zonula occludens-1) were significantly upregulated during the late stage of differentiation (Fig. 3d). Collectively, these results indicate that human SG-derived clonal cells have epithelial differentiation potential in addition to mesenchymal differentiation potential.

### *In vitro* immunosuppressive activity

One of the characteristics of MSCs is their inherent immunomodulatory or immunosuppressive activity. Thus, we examined whether the isolated human SG-derived clones have immunosuppressive properties. The immunosuppressive activity was evaluated by *in vitro* immunosuppressive assay using stimulated PBMCs. Co-culture of the clonal SG cells significantly inhibited proliferation of phytohemagglutinin-stimulated PBMC (Supplementary Fig. S1).

### Stem cell marker analysis and defining differential molecular characteristics

**Figure 4:**
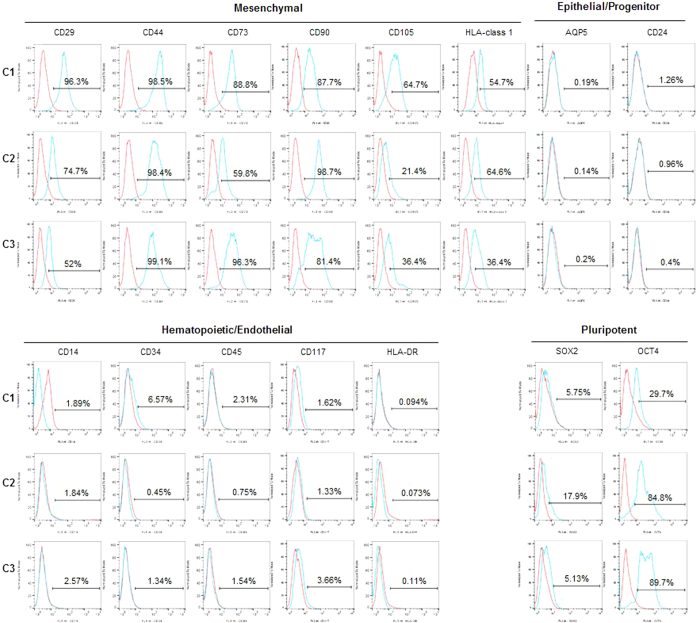
Analysis of multilineage cell marker expression. Flow cytometric analysis was conducted to monitor the marker expression of clonal SG cells. Numbers above bracketed lines indicate the percentage of the cell population. Similar to MSCs, clonal SG cells commonly expressed MSC markers, but did not express hematopoietic/endothelial markers. Clonal SG cells were also positive for Oct4, but negative for Sox2. Basal expression of AQP5 was not detected and samples were negative for CD24, a recently suggested salivary progenitor marker. Blue line, each marker indicated; red line, isotype-matched control antibody.

Next, to examine the marker expression of human SG clones, we conducted flow cytometric analysis in terms of mesenchymal, hematopoietic/endothelial, epithelial/SG progenitor, and pluripotent cell markers. All three clones were positive for mesenchymal markers (CD29, CD44, CD73, CD90, CD105, and HLA-class I), but negative for hematopoietic/endothelial markers (CD14, CD34, CD45, CD117, or HLA-DR) (Fig. 4). Specifically, they expressed pluripotent marker Oct4, but rarely expressed Sox2. Interestingly, the tentative SG epithelial progenitor markers, CD24 and AQP5, were not expressed in the clonal SG cells.

**Figure 5:**
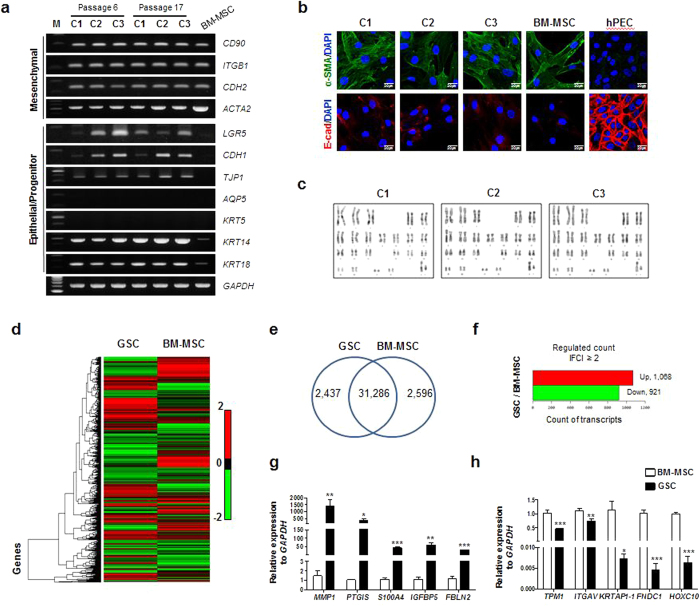
Screening differential gene expression. (**a**) Differential basal gene expression between human clonal SG cells and human BM-MSCs was analyzed by RT-PCR. The gel images are cropped for clarity from the full-length images under the same experimental conditions. The original images are provided in Supplementary Fig. 2. (**b**) Epitheliomesenchymal properties of the SG clones revealed by immunofluorescence staining for a-SMA (mesenchymal) and E-cadherin (epithelial). Primary cells isolated from human parotid glands were used as epithelial controls. Scale bars represent 20 μm. (**c**) Karyotype analysis of clonal SG cells cultured for 17 passages. (**d**) NGS-mediated transcriptome analysis comparing putative GSCs and BM-MSCs. (**e**) Venn diagram showing total number of analyzed genes for each group (GSC, BM-MSC and merged GSC/BM-MSC). (**f**) Differentially expressed genes between putative GSCs and BM-MSCs with ≥2-fold increase or ≤2-fold decrease. (**g,h**) Confirmation of NGS results for 10 genes using real-time RT-PCR.

To further analyze the differential gene expression between putative GSCs and BM-MSCs, basal mRNA expression of the selected genes was compared (Fig. 5a). Like BM-MSCs, all three clones expressed mesenchymal genes such as *ITGB1* (encoding integrin β1 or CD29), *CD90*, *CDH2* (encoding N-cadherin), and *ACTA2* (encoding a smooth muscle isoform of alpha-actin 2). Furthermore, they highly expressed epithelial genes, including *CDH1* (encoding E-Cadherin) and *TJP1*, none of which were expressed by BM-MSCs. Intriguingly, an epithelial stem cell marker, *LGR5*, was significantly expressed in the SG clones, while its expression was very weak in BM-MSCs. Salivary epithelial markers such as *AQP5* (acinar) and *KRT5* (basal/myoepithelial) were not expressed in clonal SG cells, but other transcripts such as *KRT14* (basal/myoepithelial) and *KRT18* (luminal) were detected. In addition, to exclude the possibility that phenotypic characteristics are affected by cell culture, we compared gene expression between early (p6) and late (p17) passages. Our results showed that these genes are constantly expressed during long-term serial passage, indicating that *in vitro* culture does not affect marker expression of the clonal cells (Fig. 5a).

The epitheliomesenchymal properties of SG clones were further supported by immunofluorescence staining. The SG clones were compared with BM-MSCs and human parotid epithelial cells (hPECs). As shown in Fig. 5b, co-expression of both E-cadherin (as an epithelial marker) and α-SMA (as a mesenchymal marker) was observed in SG clonal cells, whereas BM-MSCs were positive for α-SMA, but negative for E-cadherin. Moreover, hPECs highly expressed E-cadherin, but not α-SMA. We also examined the karyotypes of the clonal SG cells and found no chromosomal aberrations in long-term cultured SG clones at passage 17 (Fig. 5c).

To further characterize and understand the isolated clonal SG cells at the molecular level, we conducted transcriptome analysis between clonal SG cells and BM-MSCs using next generation sequencing (NGS) (Fig. 5d). We compared the gene expression of clonal SG cells to that of BM-MSCs. Among 36,319 transcripts, 31,286 transcripts found both in clonal SG cells and BM-MSCs were subject to analysis (Fig. 5e). Genes with a ≥2-fold increase in expression were considered upregulated, while those with a ≤ 2-fold decrease in expression were regarded as downregulated. We identified a total of 1,989 differentially expressed genes (DEGs), of which 1,068 genes were upregulated and 921 genes were downregulated in clonal SG cells relative to BM-MSCs (Fig. 5f). Some of the DEGs are listed in Supplementary Tables. The upregulated genes in clonal SG cells included *MMP1, PTGIS, S100A4, IGFBP5, CD164, CD36*, and *FGF16* (Supplementary Table S1). The most downregulated genes included *TPM1, KRTAP1-1, FNDC1, HOXC10, CDA, NRP2*, and *S1PR1* (Supplementary Table S2). Among them, we randomly selected five genes and confirmed their expression by qPCR (Fig. 5g,h). We further analyzed NGS data to support our notion that the clonal SG cells express both epithelial and mesenchymal characteristics. NGS data also indicated the epitheliomesenchymal properties of the clonal SG cells (Supplementary Table S3).

### Temporal expression pattern of some marker genes during *in vitro* differentiation into salivary epithelial cells

**Figure 6:**
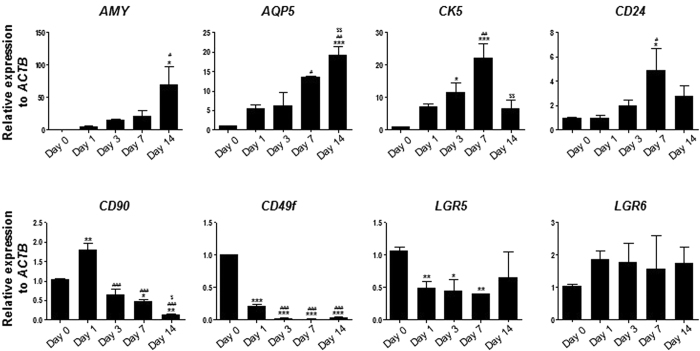
Temporal expression patterns of some genes during salivary epithelial differentiation. As a representative GSC, clone C1 was subject to salivary epithelial differentiation for up to 14 days. RNA samples were isolated at the indicated time points. Q-PCR was conducted to examine the temporal expression patterns of genes including AMY, AQP5, CK5, CD24, CD90, CD49f, LGR5, and LGR6. Results are presented as the means ± SEM. One-way ANOVA and Tukey’s post hoc multiple comparison test were performed. *, vs. Day 0; #, vs. Day 1; $, vs. Day 7. (*p < 0.05, **p < 0.01, ***p < 0.001, ^#^p < 0.05, ^##^p < 0.01, ^###^p < 0.001, ^$$^p < 0.01).

Since our data indicate the epitheliomesenchymal properties of clonal SG cells, we investigated whether salivary transdifferentiation directs the GSCs to gradually acquire epithelial phenotypes via an intermediate state of progenitor cells. To accomplish this, we examined whether our clonal SG cells as putative GSCs acquire SG progenitor or epithelial cell phenotypes during *in vitro* salivary epithelial differentiation. mRNA expression of genes including SG epithelial cell markers (*AMY* and *AQP5)*, progenitor candidate genes (*CK5* and *CD24)*, MSC markers (*CD90)*, epithelial stem cell markers (*LGR5* and *LGR6)*, and *CD49f* was evaluated by qPCR for up to 14 days of salivary epithelial differentiation (Fig. 6). As expected, both *AMY* and *AQP5 mRNAs* increased time-dependently, indicating proper salivary epithelial cell differentiation. Conversely, *CD49f* expression was switched off at all time points. We also found expression of *CK5* and *CD24* to be gradually upregulated with a peak on day 7 of differentiation. *CD9*0 mRNA was transiently upregulated at day 1 and dramatically downregulated afterward. *LGR5* expression was significantly decreased during differentiation. We also observed a statistically insignificant upregulation of *LGR6* during differentiation. Based on our results, it is likely that clonal SG cells, as putative GSCs, transiently display progenitor cell phenotypes and then acquire terminally-differentiated salivary epithelial cell phenotypes during salivary differentiation.

### Spatial localization of GSCs

**Figure 7:**
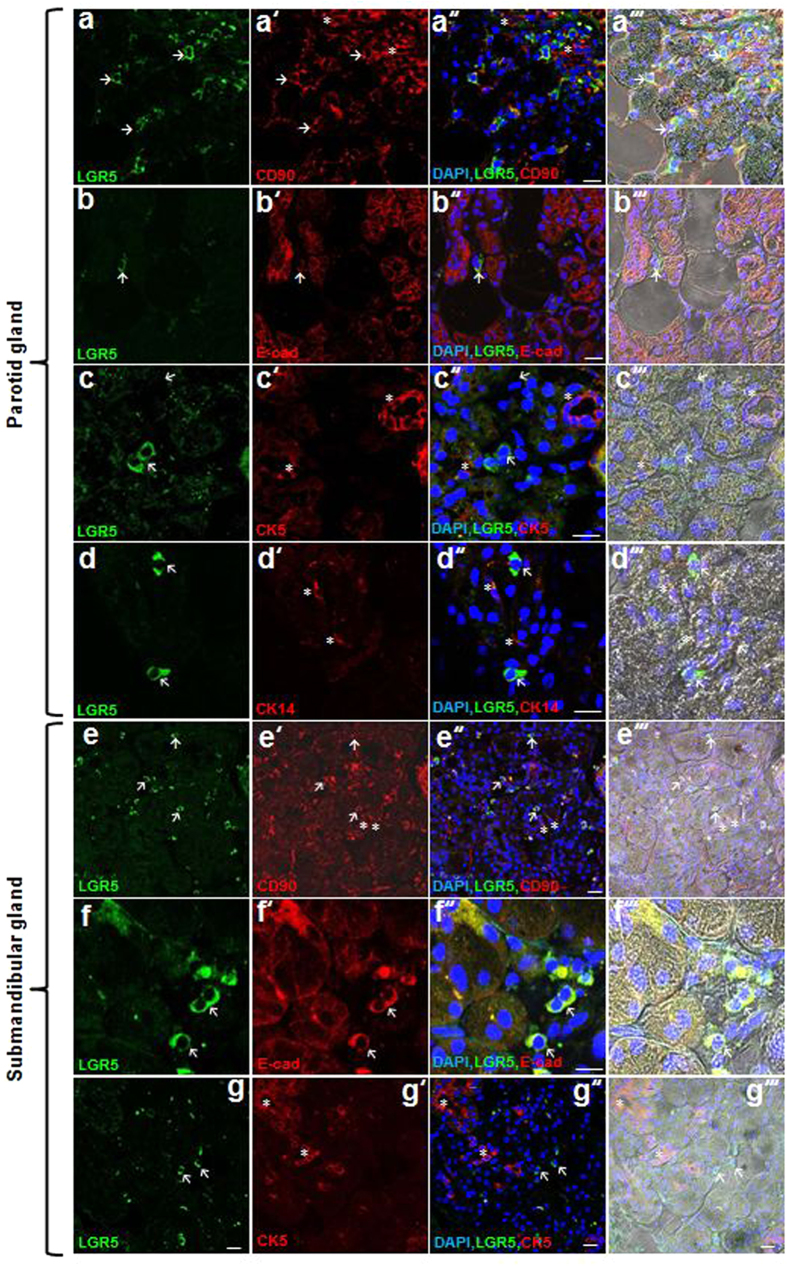
*In vivo* identification of putative hGSCs. (**a**–**g**) Patterns of LGR5 expression in human parotid glands and submandibular glands. Immunofluorescence staining shows LGR5, CD90, E-cad, CK14, and CK5 expression. Arrows and asterisks indicate LGR5^+^CD90^+^ cells and LGR5^-^CD90^+^ cells around secretory units, respectively **(a–a**′″**, e–e**′″). All LGR5^+^ cells are positive for E-cad and negative for CK14 and CK5 (**b–b**′″ and **f–f**′″, arrows; LGR5^+^E-cad^+^ cells, **c–c”’**, arrows; LGR5^+^ cells, asterisks; CK14^+^ cells, **d–d**′″ and **g–g**′″, arrows; LGR5^+^ cells, asterisks; CK5^+^ cells). All scale bars represent 20 μm.

Next, we attempted to find *in vivo* evidence of putative GSCs in human SG tissues. According to our results, we assumed that putative GSCs may co-express CD90 and LGR5. Indeed, most LGR5^+^ cells co-expressed CD90 in human SGs (Fig. 7a-a″′, e–e″′). We found that LGR5^+^CD90^+^ cells reside predominantly in inter-secretory units of human parotid glands and submandibular glands (Fig. 7a–g”’). LGR5^+^ cells co-expressed the epithelial cell marker E-cad, but not the basal/myoepithelial cell markers CK5 and CK14 (Fig. 7b–d″′, f–g″′). These results suggest that LGR5^+^CD90^+^ cells, which are putative GSCs, have properties of both epithelial and mesenchymal cells and are different from basal/myoepithelial cells.

**Figure 8:**
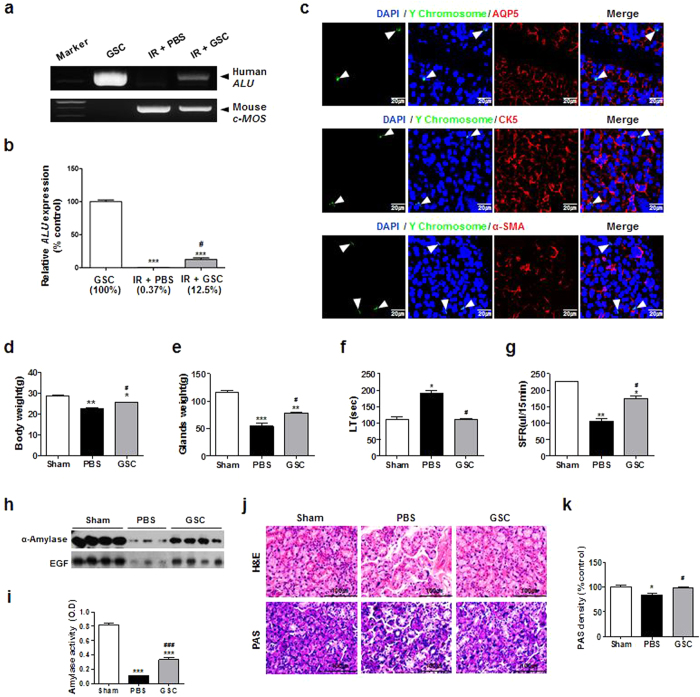
Engraftment, multilineage differentiation, and functional recovery of transplanted hGSCs in irradiated mouse salivary glands. (**a**,**b**) The level of human *ALU* sequence at 1 week after GSCs transplantation was monitored. Results are presented as the means ± SEM. *, vs. hGSC; #, vs. IR + PBS. (***p < 0.001, #p < 0.05). (**c**) FISH for human Y chromosome and immunofluorescence staining for AQP5, CK5 and α-SMA were performed with irradiated mouse salivary glands. Arrowheads indicate transplanted GSCs. All scale bars represent 20 μm. (**d**) Body weight, (**e**) submandibular glands weight, (**f**) lag time (LT) and (**g**) salivary flow rate (SFR) were measured at 12 weeks after GSCs transplantation. (**h**) Amylase activity in saliva at 12 weeks after GSCs transplantation. (**i**) Representative histological picture of H-E staining (upper) and PAS staining (lower). Scale bars represent 100 μm. (**j**) Density of PAS staining was measured using ImageJ. Results are presented as the means ± SEM. *, vs. hGSC; #, vs. IR + PBS. (*p < 0.05, **p < 0.01, ***p < 0.001, ^#^p < 0.05, ^###^p < 0.001).

We next investigated whether culture-expanded putative GSCs could be engrafted into SG tissues when transplanted *in vivo*. To accomplish this, culture-expanded clonal SG cells were locally transplanted into irradiated mouse SGs. At 1 week after transplantation, the mice were sacrificed for analysis. To identify human clonal SG cells incorporated into mouse tissues, human-specific *ALU* was amplified in genomic DNA purified from mouse SGs. Quantification of human *ALU* revealed that culture-expanded GSCs were incorporated into irradiated mouse SGs (Fig. 8a,b). To confirm these findings, we performed fluorescence *in situ* hybridization (FISH) using human Y chromosome-specific probes. A few GSCs with green fluorescent signals in the nuclei were scattered in the mouse SGs and mainly localized around salivary secretory complexes. However, FISH signals were not detected in AQP5, CK5, or α-SMA-expressing cells (Fig. 8c).

We next evaluated the therapeutic effects of GSCs *in vivo* by transplanting human GSCs into mice with irradiation-damaged salivary glands. At 12 weeks post-transplantation, GSC-transplanted mice showed improvements in both body weights and glandular weights when compared to vehicle (PBS)-injected mice (Fig. 8d,e). Salivary flow and lag time were significantly improved (Fig. 8f,g), and salivary secretory proteins of α-amylase and EGF were elevated compared to PBS-treated mice (Fig. 8h,i). Histologic examination revealed that more secretory acinar cells were preserved in GSC-transplanted mice (Fig. 8j,k). These results suggest that GSC transplantation promotes acinar regeneration, resulting in restoration of salivary secretory function damaged by radiation.

## Discussion

In this study, we established several single CFU-derived clonal GSCs from small amounts of human parotid gland biopsy through a modified subfractionation culture method. Among them, we selected three different clones and examined their stem cell properties, including cell proliferation, marker expression, and differentiation potential. Our results showed that the clonal SG cells are multipotent stem cells capable of generating mesenchymal and epithelial cell types, including salivary epithelial and hepatic cells (Figs 2 and 3). Interestingly, the clonal SG cells exhibited both mesenchymal and epithelial characteristics, suggesting that they are epitheliomesenchymal (Figs 4, 5, and Supplementary Table S3). Although basal expression of putative SG progenitor marker genes was not evident in resting clonal SG cells, the progenitor phenotypes were transiently expressed during *in vitro* differentiation into salivary epithelial cells (Fig. 6). The results of this study also provide *in vivo* evidence that LGR5^+^CD90^+^ epitheliomesenchymal cells mainly reside around human SG acnioductal structures (Fig. 7), suggesting the existence of multipotent epitheliomesenchymal GSCs in human SGs. Our findings strongly indicate that the isolated clonal SG cells are likely bona fide GSCs of human SGs.

Many researchers have attempted to identify distinct markers of SG stem/progenitor cells isolated from mouse, rat, and human SGs through various methods^12^. Either adherent cell culture or floating cell culture are commonly used to isolate SG stem/progenitor cells. Mouse salisphere-derived CD117^+^ (or c-Kit^+^) cells obtained from floating cell culture have been suggested as a potent cell therapy candidate for SG restoration after radiotherapy^9^,^13^,^14^. Recently, a mouse salisphere-derived CD117^+^ population co-expressing CD24 and/or CD49f was shown to enhance tissue repair of irradiated SGs^15^. However, Nanduri *et al*. reported that *in vivo* SG regeneration by mouse salisphere-derived CD24^+^CD29^+^ cells was not correlated with their CD117 expression^16^. Another study by Xiao *et al*. showed that the CD24^+^CD117^+^Sca1^+^ subpopulation derived from mouse salispheres augments acinar function and restores saliva secretion^17^. Conversely, murine SG stem/progenitor cells isolated by adherent cell culture have been shown to express MSC markers (CD29, CD44, CD49f, and CD90), as well as c-Kit and Sca-1^7^,^18^,^19^,^20^,^21^.

The stem/progenitor cells isolated by conventional methods generally produce a mixed and heterogeneous population. The isolated cells may readily undergo cell death or differentiation during *ex vivo* culture-expansion unless they are appropriately cultured. We recently reported a modified subfractionation culture method for rapid enrichment of single CFU-derived clonal GSCs in mouse SGs, demonstrating that mouse GSCs share MSC-like properties related to cell proliferation, immunomodulatory function, and multilineage differentiation potential^12^. In this study, we showed that our method can be applied to isolation and expansion of highly homogeneous human GSCs. Like mouse GSCs, the isolated human GSCs appeared to possess both mesenchymal and epithelial properties. However, there were discrepancies between human and mouse GSCs. For example, AQP5 was expressed in mouse GSCs, but not in human GSCs (Fig. 4).

Lombaert *et al*. showed that CD117-expressing human SG stem/progenitor cells can be isolated from salisphere cultures^9^. Others have reported that human MSC-like cells expressing various markers including CD29, CD49f, CD90, and CD105 can be obtained from adherent cell cultures^8^,^10^,^11^,^22^,^23^. Recently, Lu *et al*. isolated human minor SG-derived MSCs by the explant culture method and reported that the cells express not only MSC markers (CD29, CD44, CD73, CD90, CD105, and CD166), but also undifferentiated stem cell markers (Sox2 and nestin)^24^. However, since no unique and specific markers have been identified, further investigation is needed to define SG-specific stem cells and their distinct roles as tissue-resident stem cells.

Interestingly, human clonal GSCs isolated by our modified subfractionation culture method displayed both MSC phenotypes (CD29, CD44, CD73, CD90, CD105, α-SMA, and N-Cadherin) and some epithelial phenotypes (E-cadherin, ZO-1, and LGR5). The clonal GSCs were able to differentiate into a variety of mesenchymal and epithelial lineage cell types. NGS analysis provided further evidence that the clonal GSCs exhibited not only the epithelial phenotypes (*CLDN-3, -6, -12, KRT-8, -14, -18, -19, KLF-4, -5, TRF2, TCF3, OCLN*, and *MUC1*), but also mesenchymal phenotypes (*CD29, FN*, and *NRCAM*). The clonal GSCs also appeared to differ from SG progenitor cells. When the clonal cells were induced to differentiate into salivary epithelial cells, putative SG ductal progenitor marker genes, CK5 and CD24, were transiently expressed and peaked at 7 days post-differentiation. Moreover, LGR5^+^CD90^+^ GSCs were not co-localized with SG basal/myoepithelial cells expressing CK5 or CK14, although some transcripts of basal-type keratins were detected by RT-PCR. Taken together, our findings suggest that these cells are multipotent epitheliomesenchymal GCSs rather than lineage-committed progenitor cells. We speculate that they may regenerate various cell types in SGs

As clonal GSCs underwent salivary epithelial differentiation, gene expression of CD90, CD49f, and LGR5 significantly decreased in a time-dependent manner (Fig. 6). AQP5 and α-amylase gradually increased during differentiation. GSCs in the normal resting state did not show significant amounts of transcripts for CD24 and CK5, which is believed to be different from other SG stem/progenitor candidates^16^,^17^. Interestingly, our findings revealed LGR expression in the clonal GSCs. LGR family members, which play crucial roles in maintenance of stem cell functions, have been suggested as epithelial stem cell markers of the intestine, skin, stomach, and kidney, as well as mammary glands^25^,^26^,^27^. These findings were consistent with those a previous study that showed minor SG-derived LGR5-positive stem cells co-express some MSC markers, including CD90, CD49f, and CD29^24^.

The precise location of GSCs has been only partially understood because of the lack of known specific markers. Previous studies to identify SG stem/progenitor cells have relied on tentative markers including CD117, CK5, CK14 and Ascl3^9^,^15^,^28^,^29^,^30^,^31^. Other studies have indicated that there may be several types of progenitor cells that are usually derived from the ductal structures. For example, putative murine progenitor cells were showed to be distributed in the major secretory compartments where they were co-localized with heterogeneous populations expressing K5 and K14^32^,^33^,^34^. Another study also revealed that putative progenitor cells of adult murine SGs are preferentially found in the intercalated ducts^33^ and/or the basal layer of the lower excretory duct, with a few in the acini^34^. However, little is known about the localization of human GSCs due to lack of human GSC-specific markers. In the present study, we suggested that co-expression of LGR5 and CD90 could be used for putative identification of human multipotent GSCs. Our results indicated that there are putative GSCs in glandular mesenchyme, particularly inter-secretory units of human parotid and submandibular glands, and that most are not co-localized with CK5- or CK14-expressing basal/myopeithelial cells. To the best of our knowledge, this is the first report demonstrating the presence of multipotent epitheliomesenchymal GSCs expressing LGR5 and CD90 in human SGs. Further examination is needed to confirm the precise distribution of these GSCs through genetic lineage tracing and to understand their regenerative roles following SG damage.

## Methods

### Isolation and purification of single cell clones from human parotid gland

Human parotid gland-derived GSCs were carefully prepared from specimens of a patient who underwent parotidectomy due to benign parotid tumor with informed consent and Inha University Hospital IRB approval (permission number #2015-10-001). All experimental procedures were performed in accordance with the guidelines and regulations of Inha University. Samples were then isolated and cultured as previously described^12^. Briefly, a portion of the normal glands were resected, washed, and chopped with fine scissors and a blade, after which the minced tissue was dissociated with 0.05% collagenase II containing Hank’s balanced salt solution. The dissociated tissue solution was then filtered and centrifuged, after which the cell pellet was resuspended in Dulbecco’s modified eagle medium (DMEM; Gibco BRL, Gaithersburg, MD) containing low glucose, 20% fetal bovine serum (FBS; Gibco BRL) and 1% penicillin/streptomycin (Gibco BRL). The samples were then incubated in a 100 mm culture dish for 2 h at 37 °C under 5% CO_2_. Next, the cell culture supernatant was transferred to a new 100 mm dish and incubated for another 1 h, after which the supernatant was again transferred to a new dish (D1) and incubated for 1 h. The supernatant was subsequently transferred to another new dish (D2) and incubated for 1 day. This process was repeated two more times with 1 day incubations (D3 and D4). The single-cell derived colonies that appeared in the D2, D3, and D4 dishes were detached with 0.05% trypsin/EDTA (Gibco BRL) and isolated by cloning cylinders (Bel-Art Products, Wayne, NJ, USA), after which they were transferred to a 6-well plate and then to larger culture flasks, where they continued to expand to store a number of clonal cell populations.

In this experiment, three single cell clones (Clone 1, 2, and 3) were chosen and examined for their stem cell and molecular characteristics. As a control, single clonal bone marrow (BM)-MSCs were employed from clonal populations previously established by the same method^35^. Glandular clonal cells and BM-MSCs were cultured in DMEM (Gibco BRL) supplemented with 10% FBS (Gibco BRL) and 1% antibiotics (Gibco BRL), with the medium was replaced every 2 days during cultivation.

### Evaluation of cell proliferation and sphere-forming ability

To analyze the proliferation ability, three different established clonal cell lines (C1, C2, and C3) were seeded at a density of 3 × 10^4^ per 100 mm dish in DMEM (Gibco BRL) supplemented with 10% FBS (Gibco BRL) and 1% antibiotics (Gibco BRL). Cell numbers were counted at passage numbers 7 to 17. During subculture, cellular morphologies were observed under a light microscope (Olympus CKX41, Olympus, Tokyo, Japan) at passage 10 and 17, where cells were fixed with 10% formalin for 10 min, then stained with 0.5% crystal violet for 30 min. Doubling time was calculated by the formula (T − T_0_)Log2/logN − logN_0_, where T − T_0_ indicates culture period, N indicates cell numbers at the end of culture, and N_0_ indicates initial cell numbers.

To evaluate the sphere forming ability, cells were dissociated with 0.05% trypsin-EDTA, washed and centrifuged to remove serum, then suspended and cultured in DMEM/F12 (Gibco BRL) supplemented with 1% antibiotics, 1 × nonessential amino acids (Sigma), 1 × L-glutamine (Gibco BRL), 20 ng/ml human recombinant epidermal growth factor (EGF; R&D systems, Minneapolis, MN, USA), 20 ng/ml human recombinant basic fibroblast growth factor (bFGF; R&D systems), and 1 × N2 supplement (Life Technologies, Carlsbad, CA, USA). The cells were subsequently cultured in 1% F-127 (Sigma, St. Louis, MO, USA)-coated 35 mm Petri dishes (SPL Lifesciences Co., Gyeonggi-do, Korea) at a density of 1 × 10^5^ cells/cm^2^ and observed under a light microscope.

### *In vitro* differentiation

Three clonal cells were analyzed for their capability to differentiate into mesenchymal cells (adipogenic, osteogenic, and chondrogenic) and hepatic cell lineages as previously described^12^, and SG epithelial differentiation was further induced. Briefly, cells at passage 6 were seeded at 1 × 10^4^ cells/cm^2^ onto plates that had been precoated with matrigel (BD Biosciences), after which they were cultured in serum-free hepato-STIM medium (BD Biocoat^TM,^ Franklin Lakes, NJ, USA) supplemented with recombinant EGF (BD Biocoat^TM^), 2 mM L-glutamine, and 1% penicillin/streptomycin at 37 °C for 3 days. At the end of differentiation, cells were immunostained with anti-AQP5 antibody (Alomone Labs, Jerusalem, Israel) and anti-α-amylase antibody (Santa Cruz, CA, USA) to confirm that they were SG acinar cells. Each differentiation was further confirmed by mRNA expression of acinar cell markers as shown in Table S4.

### Flow cytometric analysis for cell surface maker expression

Established clonal cells at passage 4–9 were subjected to flow cytometry for analysis of cell surface marker proteins. Briefly, the cells were washed twice with PBS, harvested by treatment with trypsin/EDTA, then incubated with the fluorescein isothiocyanate (FITC) or phycoerythrin (PE)-conjugated antibodies. Cells were then analyzed using a FACSCalibur system (BD Biosciences, San Jose, CA, USA), after which data were analyzed using the Cell Quest software (BD Biosciences). The antibodies used for flow cytometric analysis were as follows: CD29 (BD Biosciences), CD44 (BD Biosciences), CD73 (BD Biosciences), CD90 (R&D Systems, Minneapolis, MN, USA), CD105 (BD Biosciences), and MHC-class I (BD Biosciences) for mesenchymal markers, CD14 (BD Biosciences), CD34 (BD Biosciences), CD45 (BD Biosciences), CD117 (BD Biosciences), and HLA-DR (R&D Systems) for hematopoietic markers, AQP5 (Bioss, Woburn, MA) and CD24 (BD Biosciences) for epithelial markers, and SOX2 (Cell Signaling Technology, Danvers, MA) and OCT4 (R&D Systems) for embryonic markers. Isotype-matched control antibodies were used in each antibody analysis.

### Gene Expression Analysis (RT-PCR, qPCR, NGS)

Reverse transcriptase polymerase chain reaction (RT-PCR) was performed to investigate the gene expression patterns of GSCs. Human clonal BM-MSCs were used as a control for comparison with the characteristics of MSC. Total RNA was extracted from cells at passage 6 and cDNA was synthesized from the total RNA (1 μg) using a reverse transcription system kit (Bioneer, Daejon, Korea) according to the manufacturer’s instructions. Polymerase chain reaction (PCR) was then carried out using primers specific for each gene (Table S4). PCR was conducted by subjecting the samples to 30–40 cycles of denaturation at 94 °C for 30 s, annealing at 53–60 °C for 30 s and extension at 72 °C for 30 s, after which the amplified DNA products were run on a 1.5% agarose gel and visualized by ethidium bromide staining. Images were captured using an Imaging Analyzer System (Kodak Image Station 4000R, Kodak, Rochester, NY).

During differentiation of GSCs into SG epithelial cells, the changes in marker genes of GSCs were detected by qPCR. Briefly, GSCs were harvested at 1, 3, 7 and 14 days during differentiation, after which total RNA was extracted using Trizol (Invitrogen, Carlsbad, CA). Next, 1 μg of total RNA was transcribed into complementary DNA using a PrimeScript^TM^ RT reagent kit (Takara, Shiga, Japan) according to the manufacturer’s instructions. Real-time reverse transcriptase-polymerase chain reaction (RT-PCR) was carried out using SYBR Premix Ex Taq^TM^ II containing cDNA (1 μl) and the specific primers designed for each gene sequence listed in Table S4. RT-PCR was conducted using the StepOnePlus Real-Time PCR system (Applied Biosystems, Foster City, CA). For PCR, samples were subjected to 40 cycles of denaturation at 95 °C for 10 sec and annealing and extension at 60 °C for 1 min. After amplification, a melting curve was constructed from the purified PCR products.

Total RNA of GSCs and hBM-MSCs was extracted using Trizol (Invitrogen), and then sent to Macrogen (Seoul, South Korea) for library construction and sequencing. Libraries were constructed using a TruSeq RNA Sample Prep Kit (Illumina, San Diego, CA, USA) and sequenced using a HiSeq 2000 (Illumina) to paired-end reads of 101 bp.

### Immunofluorescence Staining

Cultured cells and human tissues were examined by immunofluorescence using standard protocols. Briefly, cells were washed two times with PBS, fixed with 4% paraformaldehyde for 10 min, and then washed in PBS containing 0.05% Tween 20 (Sigma). Non-specific binding was blocked with PBS containing 1% bovine serum albumin. The cells were incubated with primary antibody for E-cadherin (R&D system) and α-SMA (Millipore, Darmstadt, Germany) in a moist chamber overnight at 4 °C. After washing in PBS, cells were incubated with Alexa-conjugated IgG (Invitrogen) for 2 h in the dark at room temperature. Next, the slides were mounted with Vectashield mounting medium with 4′,6-diamidino-2-phenylindole (DAPI; Vector Labs, Peterborough, UK). To enable comparison with salivary epithelial cells, hPECs were prepared using a previously described culture method^36^. For immunofluorescence staining of human parotid gland and submandibular gland tissues, paraffin sections were dewaxed using xylene for 30 min, after which antigen retrieval was performed by boiling in 10 mM sodium citrate for 2 min. The sections were then treated with primary antibody for LGR5 (Thermo Fisher Scientific), CD90 (Abcam), E-cadherin (R&D System), Cytokeratin 14 (Santa Cruz), or Cytokeratin 5 (Abcam) overnight at 4 °C, after which they were incubated with the secondary antibody, Alexa-conjugated IgG, for 1 h at room temperature with DAPI. All experiments included a slide with no primary antibody as a negative control. After mounting, cells were viewed using a confocal laser scanning microscope (Olympus FV1000, Olympus, Tokyo, Japan).

### Karyotyping

Karyotyping of long-term cultured GSC clone 1, 2 and 3 (passage 17) was accomplished by the chromosomal G-banding method (GenDix, Inc., Seoul, Korea).

### *In vivo* transplantation

#### Animal experiments

This study was approved by the Animal Ethics Committee of Inha University Hospital (Permit Number: 150716-371), and all experimental procedures were performed in accordance with established institutional guidelines. Six-week-old female Balb/c nude mice weighing 18–20 g were purchased from the Research Model Producing Center (Orient Bio, Seongnam, Korea). Animals were maintained under conventional clean conditions and provided with standard laboratory chow and sterilized water ad libitum. Mice were premedicated with xylazine (10 mg/kg) and anesthetized with an intraperitoneal injection of ketamine (110 mg/kg). Animals were firmly fixed in a plastic mold, after which irradiation was conducted using a 4 MV X-ray emitted from a linear accelerator (Mevatron MD, Siemens Medical Laboratories Inc., Germany) with a single dose of 15 Gy at a focus-to-skin distance of 100 cm. The animals were locally irradiated in the region of the head and neck including the salivary gland with the body shielded from the radiation field. Following irradiation, a horizontal incision of the neck was made to expose salivary glands. The GSCs were then injected (GSCs 2 × 10^5^ in 10 μL of PBS or PBS alone) to both submandibular glands of the mice using an insulin syringe with a 29-gauge needle. At 1 and 12 weeks after transplantation, saliva was collected for 15 min after stimulation by intraperitoneal injection with pilocarpine (2 mg/kg) and salivary flow rates (SFR) were calculated. Lag time was defined as the time from stimulation to the beginning of saliva secretion. After saliva was collected, body weights, submandibular glands weights and lag time were measured.

### Detection of engraftment of transplanted GSCs

PCR for human *ALU* probes was conducted to confirm the engraftment of transplanted hGSCs in recipient mouse SGs. A DNeasy^®^ Blood & Tissue Kit (Qiagen, Hilden, Germany) was used for isolation of genomic DNA of SG samples according to the manufacturer’s instructions. Primers specific for human *ALU* are described in Table S4. The amplification conditions were 95 °C for 5 min, followed by 40 cycles of 95 °C for 1 min, 55 °C for 1 min and 72 °C for 1 min, and then final extension at 72 °C for 10 min. All samples were also amplified to detect the mouse specific oncogene (*MOS*) as a control for the presence of amplifiable DNA.

### Fluorescence *in situ* hybridization (FISH)

Tissue sections were deparaffinized in xylene and rehydrated through graded alcohols to water. Slides were then incubated in sodium thiocyanate solution for 10 min at 80 °C, washed with PBS and incubated with pepsin (Sigma) for 10 min at 37 °C. Following incubation, slides were washed with PBS, and the pepsin was then quenched by submersing in glycine solution. The slides were then washed in PBS, post-fixed in paraformaldehyde solution for 2 min, washed again in PBS, dehydrated and air dried. The human centromeric Y specific probe (Cambio, Cambirdge, UK) was warmed to 37 °C, then added to slides and simultaneously denatured at 80 °C for 10 min, followed by overnight incubation at 37 °C. Following hybridization, slides were washed twice in 50% formamide wash solution (50% formamide, 50% 1 × SSC) at 45 °C for 5 min each, incubated twice in 1 × SSC at 45 °C for 5 min each and then incubated in detergent wash solution (4 × SSC, 0.5% Tween 20) 3 times for 4 min each. Hybridized probes were detected using STARFISH detection kits with FITC (Cambio). Briefly, working reagent was applied to the slide, after which it was incubated for 20 min at 37 °C and then washed 3 times for 4 min each in detergent wash solution. Immediately after washing, immunofluorescence staining was performed to confirm differentiation of transplanted GSCs. Slides were counterstained with DAPI, then viewed using a confocal laser scanning microscope (Olympus FV1000, Olympus, Tokyo, Japan).

### Histological examination

Mouse submandibular glands were fixed, paraffin-embedded, and sectioned. The samples were then stained with hematoxylin-eosin (H&E) or Periodic acid Schiff (PAS) (Diagnostic BioSystem, Pleasanton, CA, USA). PAS staining density was measured using the ImageJ software.

### Amylase activity analysis

The amylase activities of saliva secreted from salivary glands were measured using an amylase assay kit (Abcam, Cambridge, UK) according to the manufacturer’s instructions.

### Western blot analysis

Saliva samples were subjected to SDS-PAGE under reducing conditions after boiling in sample buffer at 100 °C for 5 minutes. Equal amounts of saliva were separated on 10% and 15% sodium dodecyl sulfate-polyacrylamide gels, after which the protein was transferred onto polyvinylidene difluoride (PVDF) membrane (Millipore, Billerica, MA) for 2 hours at 4 °C. The membrane was then rinsed with PBS containing 0.1% Tween 20 (PBS-T) and placed in blocking solution (5% BSA in PBS-T) for one hour at room temperature. The primary polyclonal anti-α-amylase and anti-EGF (1:1000 dilution, Santa Cruz, CA, USA) antibodies were added to a blocking solution, then incubated overnight at 4 °C. After washing in PBS-T, the membrane was incubated in horseradish peroxidase-linked goat anti-mouse IgG (1:5000 dilution, Santa Cruz, CA, USA) in blocking solution for one hour at room temperature. The membrane was thoroughly washed in PBS-T, incubated with Amersham ECL plus western blotting detection reagents (GE healthcare, Buckinghamshire, UK), and exposed to X-ray film (Agfa HealthCare NV, Mortsel, Belgium).

### Statistical analysis

Statistical analyses were conducted using the Graph Pad Prism5 package (GraphPad Software Inc., La Jolla, CA). A t-test and one-way Analysis of Variance (ANOVA) followed by Tukey’s post-hoc test were used to identify intergroup differences. A p < 0.05 was considered to indicate statistical significance.

## Additional Information

**How to cite this article**: Yi, T. G. *et al*. Single Cell Clones Purified from Human Parotid Glands Display Features of Multipotent Epitheliomesenchymal Stem Cells. *Sci. Rep*. **6**, 36303; doi: 10.1038/srep36303 (2016).

**Publisher’s note:** Springer Nature remains neutral with regard to jurisdictional claims in published maps and institutional affiliations.

## Supplementary Material

Supplementary Information

Supplementary Table S1

Supplementary Table S2
