# Supplementary material for: Single Cell Clones Purified from Human Parotid Glands Display Features of Multipotent Epitheliomesenchymal Stem Cells
Source: Sci Rep. 2016 Nov 8;6:36303. doi: 10.1038/srep36303 (PMC5099888; doi:10.1038/srep36303)
Supplement: Supplementary Information [file srep36303-s1.doc]

**Single Cell Clones Purified from Human Parotid Glands Display Features of Multipotent Epitheliomesenchymal Stem Cells**

Running title: Clonal Glandular Stem Cells from Human Parotid Glands

TacGhee Yi, PhD^1,2^, Songyi Lee, MS^2,3^, Nahyun Choi, PhD^2,3^, Hyun-Soo Shin^2,3^, PhD, Junghee Kim, MS^2^, Jae-Yol Lim, MD^2,3,*^

^1^SunCreate Co. Ltd., Yangju, Gyeonggi-do, Republic of Korea; ^2^Translational Research Center, ^3^Department of Otorhinolaryngology-Head and Neck Surgery, Inha University College of Medicine, Incheon, Republic of Korea;

* Corresponding author: Jae-Yol Lim, MD, PhD

Department of Otorhinolaryngology-Head and Neck Surgery, Inha University College of Medicine, 27, Inhang-ro, Jung-gu, Incheon 14913, Republic of Korea.

E-mail: jylim@inha.ac.kr, Tel: 82-32-890-3028, Fax: 82-32-890-3580

Figure S1. In vitro immunosuppressive activity of the SG clonal cells.

Figure S2. The original full-length gel images of the cropped images in Figure 5

Table S1. The top 50 genes upregulated in SG clone C2

Table S2. The top 50 genes downregulated in SG clone C2

Table S3. DEG (GSC/BM-MSC) obtained from NGS

Table S4. Primers used for RT-PCR


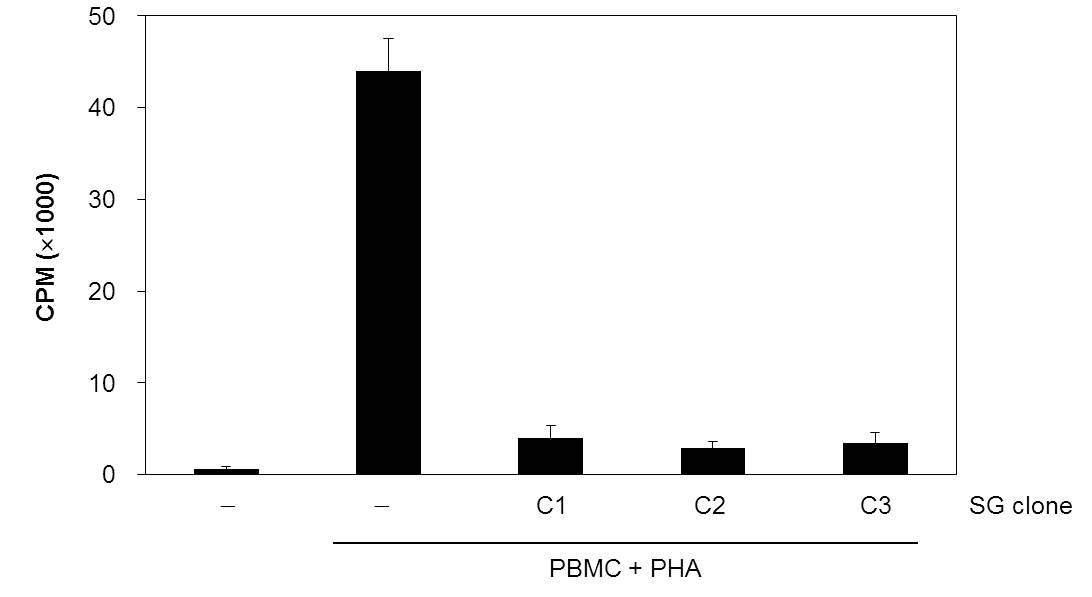


**Supplementary Figure 1. In vitro immunosuppressive activity of the SG clonal cells.**

Peripheral blood mononuclear cells (PBMC) were seeded in triplicate at a density of 2×10^5^ cells/well in a 96-well plate with or without the SG clonal cells in the presence of phytohemagglutinin (1 μg/ml). The SG clonal cells were then co-cultured with at ratios of 1:5 (SG cells: splenocytes) for 72 h. The proliferation of PBMC was determined by [3H]-thymidine incorporation for an additional 16 h. The radioactivity was measured in a beta- counter. The [3H]-Thymidine incorporation assay showed that all three SG clones significantly inhibited the proliferation of PBMC stimulated by phytohemagglutinin.


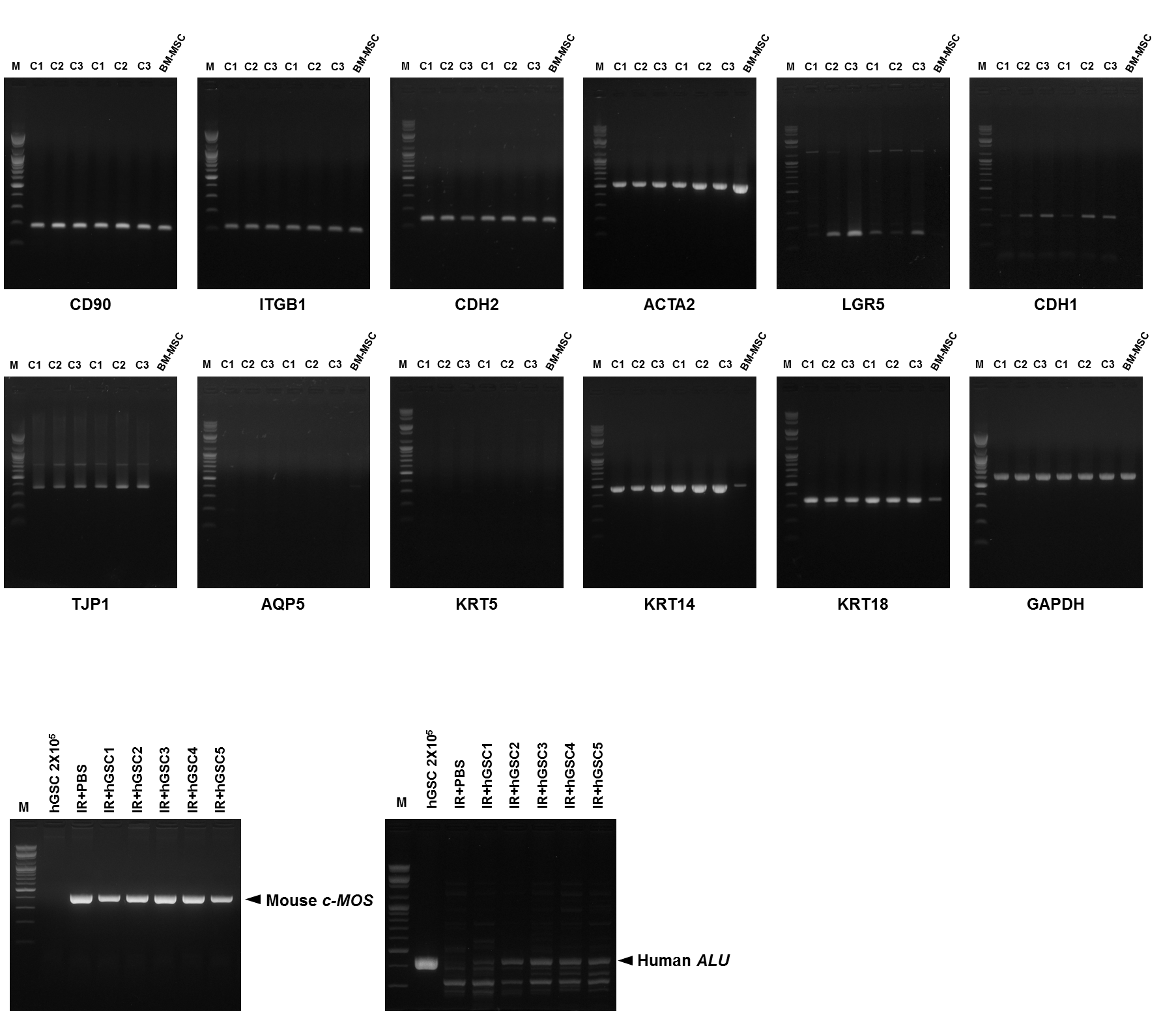


**Supplementary Figure 2. The original gel images of the cropped images in Fig. 5 .**

Supplementary Table S3

|  | DEG (GSC/BM-MSC) | Alias (Description) | Remark |
| --- | --- | --- | --- |
| mesenchymal markers | *NRCAM* (*)  *FN* (*)  *CD29* (2.7) | Neuronal cell adhesion molecule  Fibronectin  Integrin beta1 | * no expression was detected in BM-MSCs |
| epithelial markers | *CLDN3* (1.7)  *CLDN6* (1.7)  *CLDN12* (2.7)  *OCLN* (6.3)  *MUC1* (2.0)  *LGR5* (2.4)  *TRF2* (1.6)  *KRT8* (3.6)  *KRT14* (16.2)  *KRT18* (10.4)  *KRT19* (5.3)  *TCF3* (1.3)  *KLF4* (2.4)  *KLF5* (1.2) | Claudin3  Claudin6  Claudin12  Occludin  Mucin1  Leucine-rich repeat containing G protein-coupled receptor 5  Telomeric repeat binding factor 2  Keratin8  Keratin14  Keratin18  Keratin19  Transcription factor 3  Kruppel-like factor 4  Kruppel-like factor 5 |  |

Table S4. Primers used for RT-PCR

| **Gene and symbol** |  | **Primer sequences (5’-3’)** |
| --- | --- | --- |
| α-Amylase (AMY1A) | F | CATTGACATTGGCGTAGCAG |
|  | R | CAGAATGTCAAGATGGATGC |
| Aquaporin 5 (AQP5) | F | CTCTGCATCTTCTCCTCCACG |
|  | R | TCCTCTCTATGATCTTCCCAG |
| BHLHA12 (Mist1) | F | CTCCAAGATCGAGACGCTCA |
|  | R | TCTGCGTGGAGTACCTCTGC |
| ZO-1 (TJP1) | F | CGGTCCTCTGAGCCTGTAAG |
|  | R | GGATCTACATGCGACGACAA |
| E-Cadherin (CDH1) | F | CGCATTGCCACATACACTCT |
|  | R | TTGGCTGAGGATGGTGTAAG |
| N-Cadherin (CDH2) | F | AGCCAACCTTAACTGAGGAGT |
|  | R | GGCAAGTTGATTGGAGGGATG |
| Cytokeratin 5 (CK5) | F | CCCAGTATGAGGAGATTGCCAACC |
|  | R | TATCCAGAGGAAACACTGCTTGTG |
| Integrin α6 (CD49f) | F | TTGAATATACTGCTAACCCCG |
|  | R | TCGAAACTGAACTCTTGAGGATAG |
| Integrin β1 (CD29) | F | GAAGGGTTGCCCTCCAGA |
|  | R | GCTTGAGCTTCTCTGCTGTT |
| CD90 (Thy-1) | F | GACAGCCTGAGAGGGTCTTG |
|  | R | CCCAGTGAAGATGCAGGTTT |
| CD24 | F | ATGGGCAGAGCAATGGTGGCCA |
|  | R | AGAGTGAGACC ACGAAGAGACT |
| c-kit (KIT) | F | TATACAACCCTGGCATTATGTCC |
|  | R | TGCGAAGGAGGCTAAACCTA |
| Alpha smooth muscle actin (ACTA2) | F | CGATAGAACACGGCATCATC |
|  | R | CATCAGGCAGTTCGTAGCTC |
| Keratin 14 | F | TGAGCCGCATTCTGAACGAG |
|  | R | GATGACTGCGATCCAGAGGA |
| Keratin 18 | F | TGGTCACCACACAGTCTGCT |
|  | R | CCAAGGCATCACCAAGATTA |
| Leucine-rich repeat containing G protein-coupled receptor 5 (LGR5) | F | AGGATCTGGTGAGCCTGAGAA |
|  | R | CATAAGTGATGCTGGAGCTGGTAA |
| Leucine-rich repeat containing G protein-coupled receptor 6 (LGR6) | F | CCCAATCCAGTTTGTGGGAA |
|  | R | GCCTTTGAGATCTGGAAACT |
| Lipoprotein lipase (LPL) | F | TACAGGGCGGCCACAAGTTTT |
|  | R | ATGGAGAGCAAAGCCCTGCTC |
| Fatty acid binding protein 4 (FABP4) | F | CATCAGTGTGAATGGGGATG |
|  | R | GTGGAAGTGACGCCTTTCAT |
| Peroxisome proliferator activated receptor gamma 2 (PPARG2) | F | GACCACTCCCACTCCTTTGA |
|  | R | CGACATTCAATTGCCATGAG |
| Runt-related transcription factor 2 (RUNX2) | F | TATGAAAAACCAAGTAGCAAGGTTC |
|  | R | GTAATCTGACTCTGTCCTTGTGGAT |
| Bone gamma-carboxyglutamate(gla) protein (BGLAP) | F | GTGCAGAGTCCAGCAAAGGT |
|  | R | CTAGCCAACTCGTCACAGTC |
| Aggrecan (ACAN) | F | GCTACACCCTAAAGCCACTGCT |
|  | R | CGTAGTGCTCCTCATGGTCATC |
| Collagen II (COL2) | F | TTTCCCACCTCAAGATGGTC |
|  | R | TCACCTGGTTTTCCACCTTC |
| Collagen X (COL10) | F | GCCCAAGAGGTGCCCCTGGAATAC |
|  | R | CCTGAGAAAGAGGAGTGGACATAC |
| Albumin (ALB) | F | TGTTGATTGCCTTTGCTCAG |
|  | R | TGGAGACTGGCACACTTGAG |
| Matrix metallopeptidase 1 (MMP1) | F | TCGGGGCTTTGATGTACCCT |
|  | R | GGGCTGGACAGGATTTTGGG |
| Prostaglandin I2 synthase (PTGIS) | F | GACGACCACTCTCCCACAGA |
|  | R | TTGAATTCTCGCCCGTCTGC |
| S100 calcium binding protein A4 (S100A4) | F | TCTGGAGAAGGCCCTGGATG |
|  | R | CCACCTCGTTGTCCCTGTTG |
| Insulin-like growth factor binding protein 5 (IGFBP5) | F | AGAGAGACTCCCGTGAGCAC |
|  | R | TCCCCCGACAAACTTGGACT |
| Fibulin 2 (FBLN2) | F | CTCTGGGAGGTGGGAGTCAG |
|  | R | GCTGCTCAGTGACAGCCATT |
| Tropomyosin 1 (TPM1) | F | TCAGAAGGCCAAGTCCGACA |
|  | R | CCGAGTCTCAGCCTCCTTCA |
| Integrin, alpha V (ITGAV) | F | CTGTGCCTGTGTGGGTGATC |
|  | R | GTTCTTCTTGAGGTGGCCGG |
| Keratin associated protein 1-1 (KRTAP1-1) | F | TCTCAACCGGTGGGACTTGT |
|  | R | ACCTGATACGGGTGCTCACA |
| Fibronectin type III domain containing 1 (FNDC1) | F | ACAGGCGTGTGCTGATTGAG |
|  | R | TCCCAAGATGCAGCGACAAC |
| Homeobox C10 (HOXC10) | F | AGAGAAGCGGGCGAAAAGTG |
|  | R | GTAGTAGCTGGGCACGGGTA |
| Human Alu | F | CAGGACCTGAGAAAGGACACTATCC |
|  | R | CAAACAAGAGGCACACTTTCAACCA |
| Mouse-specific protooncogene c-MOS | F | GAATTCAGATTTGTGCATACACAGTGACT |
|  | R | AACATTTTTCGGGAATAAAAGTTGAGT |
| Beta-actin (ACTB) | F | AGCTGTGCTATGTTGCCCTG |
|  | R | AGGAAGCAAGGCTGGAAGAG |
| Glyceraldehyde 3-phosphate dehydrogenase (GAPDH) | F | ATGGGGAAGGTGAAGGTCG |
|  | R | TAAAAGCAGCCCTGGTGACC |
